# A systematic review of the relationship between portion size and indexes of adiposity in children

**DOI:** 10.1111/obr.13928

**Published:** 2025-04-27

**Authors:** Anca T. Dobrescu, Alice Porter, Danielle Ferriday, Peter J. Rogers

**Affiliations:** ^1^ Nutrition and Behaviour Unit, School of Psychological Science University of Bristol Bristol UK; ^2^ Population Health Sciences, Bristol Medical School University of Bristol Bristol UK; ^3^ NIHR Bristol Biomedical Research Centre University Hospitals Bristol and Weston NHS Foundation Trust and University of Bristol UK

**Keywords:** body adiposity, children, portion size

## Abstract

Research suggests that larger food portion sizes (PS) during a meal or snack increase daily energy intake in children. However, it remains unclear whether this ‘PS effect’ would lead to a sustained increase in consumption and affect body adiposity. This systematic review assesses the evidence for a relationship between served, consumed, and preferred PS (food or drink size in grams or kcal) and indexes of adiposity (e.g., BMI percentile, BMI z‐scores) in children (< 19 years). A total of 17 articles were identified using six electronic databases (PsycINFO, MEDLINE, EMBASE, Web of Science, Cochrane Library, and ProQuest Dissertations & Theses Global). Articles were screened independently by 2 researchers; 21 studies were included (15 cross‐sectional, 2 experimental, 1 case study, 1 longitudinal, and two interventions). A narrative review was conducted due to high levels of heterogeneity. Cross‐sectional and experimental studies (*n* = 13) reported positive associations between PS and adiposity, but results differed according to the sex/gender of the participants and food type. Interventions (*n* = 2) showed that portion size reduction may be effective in reducing child adiposity. Due to the limited evidence on the causality and direction of the effect, and over‐reliance on cross‐sectional studies, more evidence on the longer‐term impact of PS on indexes of body adiposity is required.

**Abbreviations**
%: percentage
Δ: mean change
ΔBMI: body‐mass index change
*β*
: standardized coefficient
adj: adjusted
B: unstandardized coefficient
BMI: body‐mass index
cal: calories
CDC: Centers for Disease Control and Prevention
CI: confidence intervals
cm: centimeters
d: days
EI: energy intake
f: female
FMI: fat‐mass index
g: grams
h: hours
IPS: ideal portion size
Kcal: kilocalories
kg: kilograms
m: male
M: mean
m^2^
: meter squared
MJ: megajoules
ml: milliliters
mos: months
MPS: maximum portion size
MS: meal size
n/N: number
NA: not available
NOS: Newcastle Ottawa Scale
NW: ‘normal weight’
OB: obese
OR: odds ratio
OW: overweight
PRISMA: Preferred Reporting Items for Systematic Reviews and Meta‐Analyses
PROSPERO: The International Prospective Register of Systematic Reviews
PS: portion size
SD: standard deviation
SDS: standard deviation scores
SE: standard error
SES: socio‐economic status
sr^2^
: squared semi‐partial correlation coefficients
UK: United Kingdom
USA: United States of America
WHO: World Health Organization
y: years

## 1 BACKGROUND

Recent data from the National Childhood Measurement Program showed that in 2022/23 more than one in four children in reception were overweight or obese (aged 4–5 years; 21.3%), and the prevalence of overweight and obesity was further increased for Year 6 children (aged 10–11 years; 36.6%).^1^ The causes of obesity are diverse and complex and involve an individual's genetics, physiology, psychology, and environment.^2^ Several dietary factors (e.g., increased energy density and changes in macronutrient composition) that may contribute to the increased prevalence of childhood obesity worldwide have been proposed.^3^ However, food PS is often discussed as a major ‘culprit’ for the obesity epidemic.^4^

Almiron‐Roig and colleagues (2018) reviewed the definitions and methods used in the wider literature regarding portion size and proposed that portion size may be defined as the amount of food consumed or served by an individual during a single eating occasion.^5^ According to their review, portion size has been typically referred to by weight (grams), volume, or household measures (e.g., tablespoons). Several portion size‐related outcomes or measures that have been discussed widely in the literature were also identified, such as the actual portion size (amount of food or drink placed on an eating utensil, such as a plate or cup, or consumed, typically measured through weighing), and ideal or desired portion size (the amount of food or drink that is served in advance of eating expected to either delay the return of hunger or achieve a certain level of fullness typically measured using online tasks). While these concepts have been commonly reported in adults, portion size estimation methods in children are even more challenging and have largely relied on dietary assessment tools, such as food photography atlases,^6^ and food records, or online dietary recall tools.^7^ Given the large variety in the definitions and methods used to estimate portion size in both children and adults, it is essential that research identifies studies that go beyond simply measuring the grams or volume of food served to or by children.

According to previous research, the PS, in grams, of most foods typically consumed by children aged two to 18 years old (e.g., salty snacks, soft drinks, fruit drinks, french fries, hamburgers, cheeseburgers, pizzas, Mexican fast foods, and hot dogs) significantly increased between 1977–1978 and 2003–2006.^8^ Additionally, Piernas and Popkin reported that during this period children's daily energy intake also increased by approximately 184 kcal. Even though most of the research investigating the availability of larger PS was carried out in the USA, similar patterns in PS trends were reported in the UK,^9^, ^10^, ^11^ and in some other European countries.^12^, ^13^ Based on this evidence, it has been argued that an increase in the availability of larger PS of common foods promoted greater energy intake, and led to a higher prevalence of overweight and obesity in children.^8^, ^14^, ^15^, ^16^

When children are served a larger PS of food, especially of more palatable energy‐dense foods, during an eating occasion (meal or snack), their energy intake increases considerably.^17^ An observation termed the ‘portion size effect’.^18^ While this is an established phenomenon, it has been largely observed in laboratory studies.^19^, ^20^, ^21^, ^22^, ^23^, ^24^, ^25^, ^26^ Fewer studies have investigated the effect of larger PS in real‐world settings, such as childcare centers, although the results appear to be consistent with those of the laboratory studies.^27^, ^28^ Nevertheless, it has been argued that laboratory paradigms where children are offered ‘free food’ may promote short‐term increases in energy intake.^29^ It is unclear whether these effects would be sustained over time because participants might reduce their food consumption over subsequent meals, days, or weeks to compensate for the previous increased energy intake.^29^

Indeed, only a small number of studies have investigated the more prolonged effects of exposing children to larger PS of food.^30^, ^31^, ^32^ For example, Smethers and colleagues (2019) observed children in childcare settings, providing them with five different daily menus of foods and beverages on two separate occasions (separated by a two‐week washout period). On one occasion, children were offered the baseline PS of the five‐day menu (100% PS condition). On the other occasion, the PS of the foods and beverages was increased by 50% (150% PS condition). The order of serving the daily menus across the five days and the order of PS conditions (100% or 150% PS) were counterbalanced. When children received the larger PS of food (50% more during meals and snacks), they consumed significantly more food at every meal, and this effect was sustained across five consecutive days. Importantly, analysis of covariance suggested that the difference in consumption between baseline and the increased PS condition was larger for children with higher sex‐specific BMI‐for‐age percentile, BMI z‐score, body weight, and height, even after adjusting for children's energy requirements. While this study indicates that the effect of larger PS on energy intake may be sustained over several days, and be influenced by measures of body size (BMI z‐score, body weight, and height), it is still unclear whether the effects of consuming larger portions would be sustained over longer time periods (i.e., would children continue to consume more from larger portions over weeks and months), and whether it would lead to significant increases in adiposity. It is also important to note that the majority of the studies that reported an increase in daily energy intake as a result of larger portions of food used food stimuli that would typically be considered more energy‐dense (e.g., macaroni and cheese).^20^ Studies that provided children with larger portions of foods lower in energy density, such as vegetables and fruits, identified an increase in consumption of these foods, but inconsistent results on energy intake depending on how these lower‐energy‐dense foods were introduced: a. if the portion size of fruits and vegetables was increased, daily energy intake also increased, b. if the portion size of other foods was decreased and the portion size of fruits and vegetables increased, daily energy intake actually decreased.^33^ Given that in most meals children will be eating foods in combination, it is important to consider the portion size of both lower‐energy and higher‐energy dense foods.

Some researchers argue that if larger PS of food is responsible for the ‘obesity epidemic’ two concerns need to be addressed: (a) whether larger PS of food has increased in popularity at a similar rate to the increase in obesity rates, and (b) whether larger PS of food is associated with an increase in adiposity.^4^, ^34^ As previously discussed, the increase in the availability of larger PS has been well documented.^5^, ^6^, ^12^, ^31^ While several studies report an association between PS and indexes of adiposity in adults and children,^35^, ^36^, ^37^, ^38^, ^39^ fewer studies investigated the longer‐term effect of PS manipulation.^40^, ^41^, ^42^, ^43^ To date, two systematic reviews and meta‐analyses have been published that investigate the effect of served PS on body weight in adults.^44^, ^45^

The first systematic review and meta‐analysis looked at the effect of served PS and ingestive frequency on body weight in adults.^44^ However, the authors did not identify sufficient studies to assess the effect of served PS on body weight. The second systematic review and meta‐analysis investigated the effect of reducing served PS on daily energy intake and body weight in adults.^45^ Four studies that investigated the effect of PS manipulation on body weight in adults were included in the review. The results of these studies suggested that when participants were allocated to being served a smaller PS of food, they gained significantly less weight (0.6 kg) compared to when they were served a larger PS. These findings are based on data from studies that manipulated PS for one or two meals for a duration that varied between four days and six months. The authors of the systematic review and meta‐analysis concluded that PS reduction may be an effective approach to reducing overweight and obesity in adults. However, neither of these reviews included studies in children. In addition, both systematic reviews only included studies that investigated the effect of PS on body weight. Obesity is typically characterized by an excess of body fat or adiposity,^46^ and central adiposity is in particular associated with worse health outcomes among children, such as increased risks of cardiometabolic problems.^47^, ^48^ Therefore, it is important to consider measures that at a minimum adjust for the height of an individual, such as BMI which can act as an indirect measure of adiposity, and ideally which can also distinguish between muscle and fat mass.

To date, one narrative review from 2021 has evaluated studies on the relationship between served food portion size and obesity in children, although only five relevant studies were identified.^49^ In both children and adults, reviews have predominantly focussed on served PS.^44^, ^45^ However, in children there is evidence to suggest that the amount of food children are served or self‐serve is typically associated with the amount of food they consume (although we note this is not strictly linear or proportional).^17^ As discussed above, where the study by Smethers and colleagues is described, we also do not yet know if larger PS served actually leads to a sustained increase in consumption (PS consumed), and subsequent adiposity. As a result, for this particular review, several portion‐size‐related outcomes are considered, such as served portion size, consumed portion size, and preferred portion size, which are defined in the section below. The aim of the current paper is to extend and further the previous evidence by systematically reviewing studies investigating the independent effect of a range of PS‐related outcomes on indexes of body adiposity in children. The review aimed to address two key questions. First, to investigate if there is an association between food PS and indexes of body adiposity in children. Second, to explore if manipulation of food PS leads to changes in indexes of body adiposity in children.

## 2 METHODS

The protocol for the current systematic review was preregistered in the international prospective register of systematic reviews (i.e., PROSPERO, Registration number: CRD42021281604). The review was conducted following the Preferred Reporting Items for Systematic Reviews and Meta‐Analyses (PRISMA) guidelines, which are presented in Supporting Information Data S2 (Table S1).^50^

### 2.1 Definitions

For the objectives of this review, we defined PS as the amount of food served or consumed at an eating occasion (meal or snack) measured in grams and/or kcal.^5^ Given the variety of definitions and methods for estimating PS, in the present review, we have used the review by Almiron‐Roig and colleagues (2018) to conceptualize some of the PS‐related outcomes of the included studies, and have reported differences in outcomes based on this. Specifically, we identified three key outcomes that were adapted for the present review: a. served portion size (the amount of food in grams or kcal served by or to children), b. consumed portion size (the amount of food typically ingested by children, either measured through direct weighing or averaged from food diaries and other measures), and c. preferred portion size (the ideal, desired, or maximum portion size selected by a child or caregiver, most likely measured using computerized tasks). In the PROSPERO pre‐registration we had initially used the term “body weight” to refer to any index of adiposity. However, upon further reflection, we decided that measures assessing adiposity are more meaningful for two reasons. First, as noted in the introduction, central adiposity can be particularly problematic in children. Second, it can be expected that larger (i.e., heavier or taller) children will eat more and gain more weight over time, because they will have, on average, higher energy requirements (comparing the energy intakes and body weights of a mouse and an elephant over time is an extreme example of this fact). Therefore, demonstrating an association (cross‐sectionally or longitudinally) between body weight and food PS is a trivial finding. As a result, in the present review, “adiposity” refers to indirect (e.g., weight‐adjusted for height, body mass index, BMI, BMI percentile) or direct indexes of body fatness (e.g., waist‐circumference, body composition) which goes beyond considering body weight (e.g., kilograms of weight). Further, studies that include only a measure of body weight (unadjusted for height) are still considered in the review (as per our registration), but are included as a positive control – we would expect that children with larger body weight (unadjusted for height) would consume larger portions of food or drink compared to children with lower body weight due to their higher energy requirements. The question remains if portion size would be associated with higher body adiposity. Evidence in ‘children’ is included in the current review, which is considered to be any individuals aged up to 19 years old (i.e., 0–18 years), consistent with the inclusion criteria of the narrative review by Flieh and colleagues (2020).^49^

### 2.2 Search strategy

Six academic databases: PsycINFO (Ovid), MEDLINE (Ovid), EMBASE (Ovid), Web of Science, Cochrane Library, and ProQuest Dissertations & Theses Global were searched. Searches combined a “portion size” term together with a “body adiposity” and a “child” term. It is important to note that we included search terms relevant to body weight, as well as body adiposity, to be as inclusive as possible, and to ensure we captured all relevant studies given that terminology related to weight and adiposity is often used inconsistently. An example of the search terms from Medline is included in Table S2 in Supporting Information Data S2. Terms were searched for in the “title” and “abstract” of the articles, for all years of records but were limited to include studies in humans and in English. Published literature and other sources such as published abstracts, trial registrations, dissertations, and theses were considered. Review articles were not included, but the reference lists were searched to identify other relevant published literature. The reference lists of all included articles were also hand‐searched for relevant articles.

### 2.3 Data management and study inclusion

All identified resources were initially transferred to EndNote before being uploaded to Rayyan (https://rayyan.qcri.org/welcome), a web and mobile application for systematic reviews.^51^ Studies were included in the current review if they reported a measure of PS, an index of body weight and/or adiposity, and an association between these measures in children aged up to 19 years old. It is important to note that studies were still considered for inclusion if they included data from participants aged over 19, as long as these represented only a small proportion of the overall sample (less than 20% of the total sample). Intervention studies were included if they explored the effect of PS manipulations on indexes of body weight and adiposity in children. Cross‐sectional or experimental studies were included if they reported: (a) an index of body weight or adiposity stratified according to PS (e.g., smaller versus larger PS of food), (b) food PS stratified according to an index of body weight or adiposity, or (c) a correlation between PS and an index of body weight or adiposity. Studies that did not report findings on the relationship between PS and an index of body weight or adiposity were excluded.

### 2.4 Screening and data extraction

All searches were performed by AD. The search results were screened for duplicates using EndNote and were removed using the automated functions. The resources were then transferred to Rayyan, where a second manual screening of duplicates was performed by AD. All results were first screened for inclusion using the titles and abstracts by two independent researchers (AD & AP). Articles were excluded only if both researchers considered them unsuitable, based on the following criteria: (a) the research was carried out in animals (non‐human) or in adults, (b) the research was not available in English, (c) the research did not include a measure of body weight/adiposity and a measure of energy intake and/or a PS‐related outcome, and (d) the article was a duplicate. Inconsistencies in decisions were resolved by a third reviewer (PR or DF). All potentially relevant articles were subsequently screened for appropriateness via full text by AD and AP. At this stage of the review, studies were excluded if: (a) there was no quantitative assessment of food PS or body weight/adiposity, (b) there was no report of the relationship between food PS and body weight/adiposity, (c) the observation or intervention was confounded by other variables, such as eating rate, (d) the article was a review of evidence, (e) the article was a duplicate, (f) the research was in adults, and (g) the research was not available in English. Study authors were contacted if more information was required to decide whether the study should be included. If the authors did not respond to requests for further information, and the identified resource did not provide sufficient information about the study to be able to assess its relevance for the current review, the identified resource was excluded. Data from included papers were extracted by AD using a pre‐piloted spreadsheet. The second reviewer (AP) independently extracted data from a proportion of the included articles (10%) using a copy of the pre‐piloted spreadsheet. Extracted data were compared between reviewers to reduce bias and potential error. There were minimal differences between the two reviewers, which were addressed through discussion. As a result, it was concluded that no further duplicate data extraction was necessary. All data extracted from the included articles are available in the Supporting Information (see Data S1).

Descriptive information for each study (e.g., title, authors, year of publication), sample characteristics (e.g., sample size, age, sex/gender), study characteristics (e.g., study design, statistical analyses), and information about the outcomes of the research (measures of food PS and body adiposity) were extracted. Consistent with procedures reported in a previous review,^45^ where data relating to the outcomes were not numerically reported in the article, data were extracted from figures using the WebPlotDigitizer (https://apps.automeris.io/wpd/). Additionally, information required for the assessment of the risk of bias using the Newcastle‐Ottawa Quality Assessment Scale (i.e., NOS) was obtained.

### 2.5 Risk of bias assessment

Risk of bias was assessed using an updated version of the NOS, which was previously used to assess bias in cohort studies (see Supporting Information 4).^52^, ^53^ This scale assesses three main areas; selection of the sample, comparability of study analysis, and the ascertainment of the outcome, and it is the most commonly used tool to assess risk of bias in cohort studies.^54^, ^55^ Additionally, based on the risk of bias indicators used in a previous systematic‐review, the conflicts of interest and funding statements were evaluated together with the availability of pre‐registrations for the included studies.^45^

### 2.6 Data synthesis and analysis

Studies were organized according to their design (association studies, interventions) and the research question. In the results tables and figures, studies are ordered based on the date of publication (starting with the most recent). Due to the high heterogeneity in the measures of PS‐related outcomes and weight/adiposity, and the limited number of prospective and intervention studies a meta‐analysis was not performed. Instead, a narrative synthesis was conducted using the selected studies. Nonetheless, a measure of effect size (Cohen's *d*) was calculated where sufficient data was available. Online effect size converters (https://www.psychometrica.de/effect_size.html; https://www.escal.site/) were used to estimate Cohen's *d* for included studies, by following recommendations and guidance from previous literature.^56^, ^57^ In certain cases, obtaining Cohen's *d* was more challenging due to the lack of sufficient data. To maximize the effect size calculations, solutions were identified based on recommendations from published articles. For example, when only standardized coefficients were reported and extracted for an included study, the standardized coefficients were first converted into a correlation coefficient (*r*), and then were converted into Cohen's *d*. This followed recommendations from Peterson and Brown (2005) who suggested that it is possible to obtain a correlation coefficient (*r*) and then Cohen's d from standardized coefficients (β), if standardized coefficients are within the following range [−0.5, +0.5].^58^

## 3 RESULTS

**FIGURE 1:**
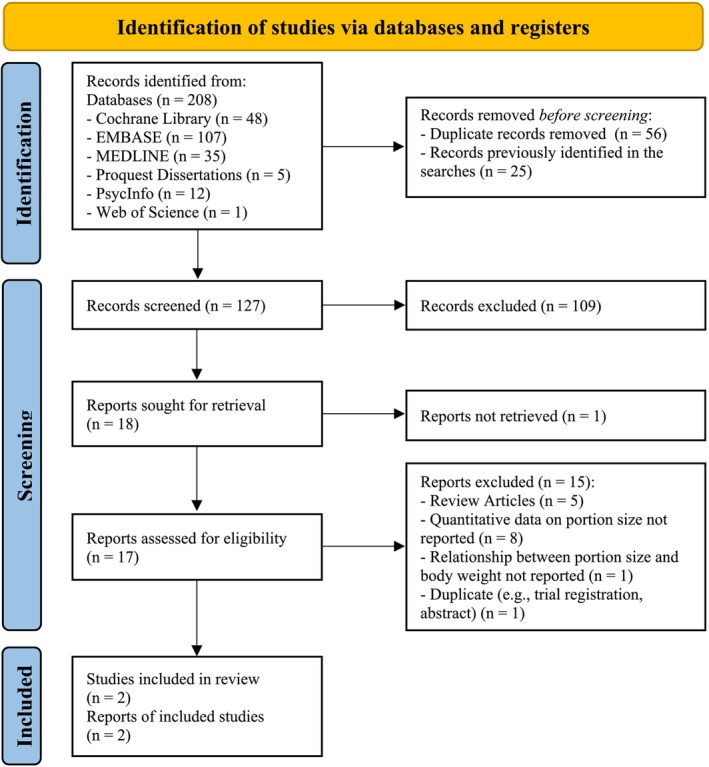
PRISMA flow diagram showing the identification of resources for the systematic review for the first search. The PRISMA flow diagrams for the subsequent searches are included in Supporting Information Data S2, Figure S1 and Figure S2.

The searches were initially performed on 30 June 2021. In the first search, a total of 2,104 studies were identified, but 1,367 remained after duplicate removal. A total of 1,227 studies were excluded in the first stage of the review, using the title and abstract (see Figure 1 for a breakdown of the exclusion criteria). From the 140 articles that were sought for full‐text retrieval, the authors could not identify the full‐text of five articles; two of the articles (a case study and a book chapter) and a thesis were not publicly available and could not be obtained through inter‐library loans, and two were conference abstracts. For the latter two articles, we attempted to contact authors to obtain full‐text but received no response. While 135 articles were assessed for eligibility using full‐text, only 19 studies were considered suitable for inclusion. Upon searching the reference lists of the excluded reviews, one additional relevant paper was identified, which led to a total of 20 studies being eligible for review (Figure 1 outlines each stage of resource identification). Later in the data extraction, authors identified a duplicate (published abstract)^59^ of a journal article which was already included for review,^60^ therefore, the abstract was removed from further analyses and 19 studies remained. The searches were updated on 18 August 2022 where no further studies were identified (see Figure S1 in Supporting Information Data S2), and again on 10 April 2024. In 2024, two studies met the inclusion criteria (see Figure S2 in Supporting Information Data S2), leading to a total of 21 studies being included in the review.

### 3.1 Characteristics of studies

The included articles were published between 1980 and 2023 (see Tables 1 and 2 for more characteristics of the studies). The earliest research study was published in 1980,^75^ while three studies were published in early 2000.^37^, ^43^, ^74^ The rest of the studies were published between 2009 and 2024.^35^, ^42^, ^59^, ^60^, ^61^, ^62^, ^63^, ^64^, ^65^, ^66^, ^67^, ^68^, ^69^, ^70^, ^71^, ^72^, ^73^, ^76^ To note, one of the publications included in the current study was a thesis,^74^ and four publications were conference abstracts.^61^, ^62^, ^72^, ^76^ Data were available from 16 countries, and research was predominantly conducted in the USA,^37^, ^43^, ^60^, ^66^, ^74^, ^75^ and in European countries, such as Germany,^69^ United Kingdom,^35^, ^61^, ^64^, ^68^, ^70^ and France.^42^, ^67^ One of the included articles was a multi‐center study which included data from eight European countries (Germany, France, Italy, Sweden, Austria, Spain, Belgium, Greece).^49^ Fewer studies were conducted in Asia^62^, ^65^, ^71^, ^73^, ^76^ and South America.^72^

**TABLE 1:** Characteristics of association studies investigating the relationship between PS and a measure of body weight/adiposity in children.

Reference	Study population	PS measure	Adiposity outcome	Key findings
Coxon et al, 2023^61^	229 children and adolescents from the UK aged between 8 and 20 y	Maximum Portion Size (MPS, preferred PS) measured on computerized task with images of varying PS of five foods	BMI z‐scores Relative fat mass index (FMI) Anthropometric data recorded under the supervision of researchers	BMI z‐scores & FMI **were not significantly correlated** with MPS in children and adolescents: zBMI & peas: *r* = 0.1, *d* = 0.2 zBMI & sweetcorn: *r* = 0.09, *d* = 0.18 zBMI & peanuts: *r* = 0.11, *d* = 0.22 zBMI & chocolate: *r* = 0.13, *d* = 0.26 zBMI & M&Ms: *r* = 0.09, *d* = 0.18 FMI & peas: *r* = −0.07, *d* = − 0.07 FMI & sweetcorn: *r* = − 0.1, *d* = − 0.2 FMI & peanuts: *r* = − 0.034, *d* = − 0.06 FMI & chocolate: *r* = 0.03, *d* = 0.06 FMI & M&Ms: *r* = − 0.05, *d* = − 0.1 No **significant** associations between FMI & MPS in multiple regression models (adj for age, time since last meal and eating rate) MPS of peas: *B* = − 0.99 MPS of sweetcorn: *B* = −2.72
Yamada, 2023^62^	63 Japanese children aged between 4 and 6 y	Actual PS consumed by children measured by weighing the lunch box before and after consumption	BMI (kg/m^2^)	PS was **significantly associated** with BMI, but only in the older class (aged 5–6 y) and in children with normal BMI.
Flieh et al., 2021^63^	1421 European children (53.5% f & 44.9% m) aged between 12.5 and 17.5 y (*M* = 14.7 ± 1.2)	Average PS (g) consumed, self‐reported by completing a 2‐day 24‐h recall computerized task	BMI (kg/m^2^) Weight Status determined using adult obesity cut‐off points FMI (body fat mass kg/m^2^) Anthropometric data recorded by researchers	**Positive and negative associations** between PS and weight status, BMI, and FMI were identified but varied according to the gender of the participant and food type. Only significant findings in plausible reporters are reported below: **PS & weight status ordinal logistic regression (adj for age, physical activity, total EI, and SES):** Bread and Rolls (f only): OR = 1.002, 95% CI (1.000, 1.004), *p* = 0.012, *d* = 0.001 Sweet Bakery Product (f only): OR = 0.996, 95% CI (0.991, 0.999), *p* = 0.046, *d* = 0.002 Carbonated soft and isotonic drink (m only): OR = 1.001, 95% CI (1.000, 1.002), *p* = 0.032, *d* = 0.001 **PS & BMI multiple linear regression (adj for age, physical activity, total EI and SES):** Breakfast Cereals (m only): *β* = 0.012, 95% CI (0.000, 0.023), *p* = 0.048, *d* = 0.12 Sweet Bakery Product (f only): *β* = − 0.004, 95% CI (−0.008, −0.001), *p* = 0.014, *d* = − 0.11 **PS & Fat mass index multiple linear regression (adj for age, physical activity, total EI and SES):** Sweet Bakery Product (f only): *β* = − 0.006, 95% CI (−0.010, −0.002), *p* = 0.005, *d* = − 0.11
McGale et al, 2020^64^	41 children from the UK (61% f, 90% white) aged between 7 and 11 y (*M* = 9.0 ± 1.5 y)	Actual PS (g) self‐served and consumed by children measured by weighing the grams of food discreetly	BMI z‐scores calculated using WHO guidelines Anthropometric data recorded by researchers	BMI z‐score was **not associated** with PS served and consumed of cereal or total meal size: BMI and cereal served: *F* (1,31) = 0.11, *p* = 0.74, *d* = 0.106 BMI and cereal consumed: *F* (1,31) = 0.32, *p* = 0.58, *d* = 0.17 BMI and total meal consumed (including milk): *F* (1,31) = 0.21, *p* = 0.65, *d* = 0.14
Mahfida et al, 2019^65^	82 mother‐toddler dyads from Indonesia; Toddlers (46% f) were aged between 9 and 60 months	Estimated PS (consumed) reported by mothers using scaled food pictures questionnaire by food groups.	Body Weight Weight status determined using weight for age, length or height for age, weight for length or height based on WHO guidelines Anthropometric data recorded by healthcare professionals	**Positive associations** between PS of overall foods, PS of vegetables, PS of sources of animal protein, and child body weight: Overall Food PS & Body Weight *p* = 0.0011 Vegetables PS & Body Weight *p* = 0.007 Sources of Animal Protein PS & Body Weight *p* < 0.001 **No association between PS** (overall foods or based on foods type) and indexes of weight status (weight for age, length or height for age, and weight for length or height).
Tripicchio et al., 2019^66^	6545 adolescents (50.3% f, 57.7% non‐Hispanic white) aged between 12 and 19 y (*M* = 15.4 ± 0.05 y) from the USA	Average PS (kcal) consumed, self‐reported by completing 2‐day 24‐h dietary recalls	Weight status determined using BMI percentiles and CDC growth charts Anthropometric data recorded by trained staff	PS (kcal) of snacks was positively **associated** with weight status: **Weight status & PS of beverages & foods (adj for snacking frequency and energy density of snacks, *n* = 6545):** NW (*n = 4062*): *M* = 262.32, *SE* = 4.41; OW (*n = 1097*): *M* = 305.41, *SE* = 8.84, *p* < 0.001, *d* = 0.15; OB (*n = 1390*): *M* = 339.60, *SE* = 10.12, *p* < 0.001, *d* = 0.23; **Weight status & PS of foods only (adj for snacking frequency and energy density of snacks, *n* = 6223):** NW: *M* = 184.78*, SE* = 3.61; OW: *M* = 213.01, *SE* = 9.17, *p* = 0.003; OB: *M* = 253.68, *SE* = 17.14, *p* < 0.001; **Weight status x PS of beverages only (adj for snacking frequency and energy density of snacks, *n* = 4736):** NW: *M* = 163.68, *SE* = 5.2; OW: *M* = 176.35, *SE* = 6.38, *p* = 0.12; OB: *M* = 205.51, *SE* = 12.45, *p* < 0.006;
Godefroy et al, 2018^67^	414 French children (46.7% f) aged between 8.08 and 11.92 y (*M* = 10 ± 1.15)	Actual PS consumed, self‐reported by selecting from 7 images with varying PS of 10 foods	BMI‐for‐age and sex Anthropometric data reported by parent	Positive **significant** association between PS and BMI‐for‐age and sex according to standardized path coefficients: PS & BMI for age and sex: *β* = 0.14, *p* ≤ 0.001, *d* = 0.38
Potter et al, 2018^68^	203 parent–child dyads from the UK; Children (59% f) were aged between 5 and 11 y (*M* = 8.1 ± 1.8)	Ideal and Maximum Portion Size (IPS, MPS, preferred PS); PS (kcal) measured on PS computerized task, with images of varying PS of seven main meals Parent‐report of child IPS & MPS Child‐report of IPS & MPS	BMI percentile computed using the CDC online calculator Anthropometric data recorded by researchers	Child IPS and parent‐report of child IPS and MPS were **significantly correlated** with child BMI percentile. Although parent‐report of IPS and MPS **best‐predicted** child‐BMI percentile. **Pearson Correlations (*r*):** Child IPS & Child BMI percentile: *r* = 0.13, *p* < 0.05, *d* = 0.26; Child MPS & Child BMI percentile: *r* = 0.006, *p* > 0.05, *d* = 0.012; Parent‐report of Child IPS & Child BMI percentile: *r* = 0.39, *p* < 0.001, *d* = 0.847; Parent‐report of Child MPS & Child BMI percentile: *r* = 0.30, *p* < 0.001, *d* = 0.629; **Multiple regression:** **Model 1 (Child IPS, Parent IPS, Parent‐report of child IPS, Parent BMI, Liking):** *R* ^*2*^ = 0.24, *R* = 0.49, *p* < 0.001 Child IPS: *B* = 0.005, *β* = 0.03, 95% CI (−0.016, 0.027), *sr* ^*2*^ = 0.001, *d* = 0.002; Parent‐report of Child IPS: *B* = 0.074, *β* = 0.33, 95% CI (0.046, 0.103), *sr* ^*2*^ = 0.1, *d* = 0.2; **Model 2 (Child MPS, Parent MPS, Parent‐report of child MPS, Parent BMI, Liking):** *R* ^*2*^ = 0.19, *R* = 0.43, *p* < 0.001 Child MPS: *B* = − 0.009, *β* = − 0.07, 95% CI (−0.028, 0.009), *sr* ^*2*^ = 0.003, *d* = −0.006; Parent‐report of child MPS: *B* = 0.054, *β* = 0.27, 95% CI (0.028, 0.080), *sr* ^*2*^ = 0.07, *d* = 0.014;
Torbahn et al, 2017^69^	131 German adolescents with obesity (52% f), aged between 8 and 16 y (M = 11.78 ± 1.78 y)	Ideal PS (preferred PS); Parent‐reported pasta PS assessed on a linearly ascending scale from 1 to 7	BMI‐SDS (adj for age and sex) reduction at 1‐y and 2‐y	PS **positively correlated** with 1‐y & 2‐y BMI‐SDS reduction: PS & 1‐y BMI‐SDS reduction: *r*_*s*_ = 0.23, *p* = 0.01, *d* = 0.472 PS & 2‐y BMI‐SDS reduction: *r*_*s*_ = 0.18, *p* = 0.04, *d* = 0.366
Syrad et al, 2016^70^	1939 children from the UK (96% white British and 52% f) aged 21 months to 5 y (age at baseline *M* = 24.35 ± 1.02 months)	Average meal size (MS) consumed; 3‐day parent‐reported diet diaries completed at 21 months	‘Growth’ represented by weight gain (kg and % growth per week)^a^	MS **positively associated** with growth rate (adj. For sex, gestational age, birth weight, the difference in age between diary completion and weight measurement): MS (10 kcal/eating occasion) & ‘Growth rate’: *B* = 1.5, SE = 0.5, growth increase = 4.0, *p* = 0.005
			Weight at baseline Weight Status defined using weight SDS^b^	MS **positively associated** with weight at baseline and risks of being overweight (adj for child sex, gestational age, birth weight, and difference in age between diet diary completion and weight measurement): MS & Weight at baseline: *B* = 21, SE = 7, *p* = 0.002 MS & Risks of overweight^c^: OR = 1.04, 95% CI (1.01; 1.07), *p* = 0.006, *d* = 0.022
ALFaris et al, 2015^71^	127 adolescent Saudi girls aged between 13 and 18 y	Ideal PS self‐reported (preferred PS) by completing descriptive questionnaire with three reference fast food PS (small, medium, large)	Waist and hip circumference (cm) Anthropometric data recorded by researchers	Adolescent girls who usually ate larger PS of fast food had **significantly** higher mean waist circumference (*p* = 0.006) and mean hip circumference (*p* = 0.001) **Means from** Figure 1 **(A & B):** Small (regular) PS: waist circumference (*M* = 69.96 cm) & hip circumference (*M* = 94.09) Medium PS: waist circumference (*M* = 72.89 cm) & hip circumference (*M* = 93.92 cm) Large PS: waist circumference (*M* = 79.25 cm) & hip circumference (M = 105.68 cm)
Albar et al, 2014^35^	636 British adolescents (48.4% f, 88.2% white) aged between 11 and 18 y (*M* = 14.6)	Average PS (g/ml) consumed, calculated from 4‐day food diary Reported by parent if child < 12 y	BMI (kg/m^2^) classified using UK 1990 reference values Anthropometric data recorded by trained staff	The PS of some foods was **associated** with BMI change in normal reporters. **PS & BMI‐change in normal reporters (adj for chid age, sex, and misreporting, *n* = 171):** Biscuits PS (10 g, *n* = 118): ∆BMI = 0.283, 95% CI (0.01, 0.56), *p* = 0.04 Buns, Cakes, and Confectionery PS (10 g, *n* = 118): ∆BMI = 0.185, 95% CI (0.05, 0.33), *p* = 0.01 Cheese PS (10 g, *n* = 118): ∆BMI = 0.258, 95% CI (0.04, 0.52), *p* = 0.05
Cabrera et al, 2013^72^	102 Cuban scholars (51 OW and 51 NW) paired by age (*M* = 8 ± 1.0), sex, and SES	Average PS consumed, calculated using a semiquantitative food frequency questionnaire	BMI Height and weight measured to calculate BMI	PS of some foods was **significantly** larger in OW children and was **highly associated** with BMI. PS of sausages (*p* = 0.006), canned fruits (*p* = 0.02) and cereals and tubers was larger in OW children. PS of sausages (*p* = 0.015), rice (*p* = 0.002), and wheat flour (*p* = 0.004) was highly associated with BMI.
Lin et al, 2013^73^	1138 nursery children (47% f) aged between 3.1 and 6.7 y (*M* = 4.84 ± 0.77) from China	Actual PS (g) consumed by children measured by weighing the grams of food discreetly	Weight Status determined using 2000 USA Growth Charts Anthropometric data recorded by healthcare professionals or researchers	Consumption of increased PS of rice, dishes, and total food **was positively associated** with child weight status: **PS & Weight Status (adj for child age & sex, mother's education, family income, and clustering within kindergartens):** Increased PS of rice & Weight Status: *OR* = 2.274, 95% CI (1.360, 3.804), *p* = 0.001, *d* = 0.45; Increased PS of dishes & Weight Status: *OR* = 1.378, 95% CI (1.010, 1.881), *p* = 0.04, *d* = 0.17; Total increased PS & Weight Status: *OR* = 1.390, 95% CI (1.109, 1.741), *p* = 0.004, *d* = 0.18
Savage et al, 2012^60^	63 children (58.7% f) aged between 3 and 5 y (average ~ 4 y) from USA	Actual PS (g) served during an ad libitum lunch, measured by weighing the grams of food discreetly	BMI z‐scores & Weight Status determined using CDC guidelines Anthropometric data recorded by trained staff	BMI z‐score **was not a significant** predictor of self‐served PS: PS & BMI z‐score (**adj for responsiveness to PS**): *R* ^*2*^ = 0.06, p < 0.01, *β* = 0.17, *d* = 0.5; PS & Weight Status were **positively associated:** PS & Weight Status: Non‐OW (*n* = 46, approximated from Figure 2.A) *M* = 244, *SE* = 14; OW (*n* = 17, approximated from Figure 2.A) *M* = 325, *SE* = 21, *p* < 0.01, *d* = 0.89;
Lioret et al, 2009^42^	719 French children aged between 3 and 11 y	Average PS (g) consumed, calculated from 7‐d diaries using PS pictures from a food manual. Reported by parent if child < 10 y	Weight status determined using IOTF age‐ and gender‐specific BMI (kg/m^2^) cut‐off points Anthropometric data reported by parent if child < 10 y	PS of biscuits and sweetened pastries was **positively associated** with OW in 3–6 y children. PS of liquid dairy products **was positively** associated with OW in 7–9 y children. **PS & OW in 3–6 y children (*n* = 329)** Biscuits (adj for child sex & age): TI (non‐consumers) *OR* = 1.00; T2 *OR* = 1.47, 95% CI (0.68–3.17), *d* = 0.21; T3 *OR* = 2.20, 95% CI (1.03–4.68), *d* = 0.43; Trend *p* = 0.039 Sweetened pastries (adj for child sex, age, dietary energy density, leisure time physical activity & sedentary behavior): T1 *OR* = 1.00; T2 *OR* = 1.38, 95% CI (0.56–3.40), *d* = 0.17; T3 *OR* = 2.99, 95% CI (1.31–6.48), *d* = 0.60; Trend *p* = 0.005 **PS & OW in 7–11 y children (*n* = 390)** Liquid dairy products (adj for child sex, age, dietary energy density, leisure time physical activity & sedentary behavior): T1 *OR* = 1.00; T2 *OR* = 0.57, 95% CI (0.29–1.11), *d* = 0.3; T3 *OR* = 0.30, 95% CI (0.14–0.60), *d* = 0.66; Trend *p* = 0.0006
Huang et al, 2004^37^	1995 children (49.6% f, 80% White) aged between 3 and 19 y (*M*_male_ = 10.9 ± 0.2; *M*_*f*emale_ = 10.6 ± 0.2) from USA	Average PS (g) consumed, calculated from 2‐day diet diaries; Reported by parent if child < 12 y	BMI percentile determined using USA growth charts Anthropometric data reported by parent if child < 12 y	**No significant association** between PS and BMI percentile in 3–8 y boys and girls, and 6–11 y girls. **Positive associations** between PS and BMI percentile in 6–11 y boys and 12–19 y boys and girls (**adj for ethnicity, percentage above poverty, urbanicity, geographic region, and daily TV viewing hours**): PS (g) & BMI percentile in boys 6–11 y (*n* = 250): *β* = 0.050, *SE* = 0.0018, *p* = 0.01, *d* = 0.2; PS (g) & BMI percentile in boys 12–19 y (*n* = 201): *β* = 0.036, *SE* = 0.010, *p* < 0.001, *d* = 0.17; PS (g) & BMI percentile in girls 12–19 y (*n* = 180): *β* = 0.034, *SE* = 0.007, *p* = 0.001, *d* = 0.17;
Rodgers, 2004^74^	45 Native American children (46.7% f) aged between 10 and 12.6 y (*M* = 11.3 ± 4.6)	Ideal PS (g, preferred PS) of 5 foods which children selected from 3 PS available (small, medium, large)	BMI z‐score calculated using CDC 2000 growth charts Anthropometric data recorded by staff	**No significant correlations** between PS (g) & BMI for age z‐score: Hamburgers: *r*_*s*_ = 0.12, *p* > 0.05, *d* = 0.24 Carrots: *r*_*s*_ = 0.03, *p* > 0.05, *d* = 0.06 Soda pop: *r*_*s*_ = 0.03, *p* > 0.05, *d* = 0.06 Potato chips: *r*_*s*_ = − 0.06, *p* > 0.05, *d* = − 0.12 Orange juice: *r*_*s*_ = 0.02, *p* > 0.05, *d* = 0.04
McConahy et al, 2002^43^	899 children (72% Caucasian) aged between 1 and 2 y from USA	Average PS (g) z‐score consumed, calculated from 2‐day dietary recalls recorded by researchers during home visits.	Body weight percentile calculated using CDC 2000 growth charts Anthropometric data reported by survey respondents	PS z‐scores were **positively associated** with body weight percentile: **Mean PS z‐score from** Figure 1: Body weight < 15th percentile: *M* = − 0.145; Body weight 15th–85th percentile: *M* = 0.005; Body weight > 85th percentile: *M* = 0.16; *p* < 0.05
Waxman et al, 1980^75^	8 children (4 classified with OB, 100% m) aged between 4.5 and 13 y from USA	Average PS (cal) consumed observed during 4 eating occasions	Weight Status (based on weight only) determined using the Wetzal Grid & 1977 Vital Health Statistics growth curve Anthropometric data recorded by researchers	The siblings with OB consumed **significantly** larger PS compared to their non‐OB siblings and their non‐OB peers. **PS & body weight status with siblings (adj for height):** OB: *M* = 766, *SD* = 290 (cal) non‐OB: *M* = 504, *SD* = 183 (cal) *F* (1,33) = 23.42, *p* < 0.001, *d* = 1.081 **PS & body weight status with peers (adj for height):** OB: *M* = 901, *SD* = 217 (cal) non‐OB: *M* = 500, *SD* = 386 (cal) *F* (1,9) = 29.58, *p* < 0.001, *d* = 1.46

This table describes characteristics of the association studies included in the review which investigate the relationship between an index of portion size and a measure of body weight/adiposity in children. Information about the study population, the type of portion size measure, adiposity outcome, and key findings from 20 studies is included. The studies are presented in descending order based on publication date, for example, the study by Coxon and colleagues (2021) is presented first as it is the most recent. A note is included at the bottom of the table to clarify abbreviations used in the table.

Note. Abbreviations: f, female; m, male; y, years; M, Mean; PS, portion size; g, grams; h, hours; BMI, body mass index; kg, kilograms; m^2^, meter squared; FMI, fat mass index; adj, adjusted; EI, energy intake; SES, socio‐economic status; OR, odds ratio; CI, confidence intervals; *β*, standardized coefficient; WHO, world health organisation; %, percentage; USA, United States of America; kcal, kilocalories; h, hours; CDC, Centers for Disease Control and Prevention; SE, standard error; IPS, ideal PS; MPS, maximum PS; B, unstandardised coefficient; sr^2^, squared semi‐partial correlation coefficients; MS, meal size; cm, centimeters; ml, milliliters; ∆BMI, BMI change; OW, overweight; NW, ‘normal weight’; T1–3, tertile 1, 2, and 3 of the PS distribution of food groups, which were determined by splitting the population into three classes, the first corresponding to non‐consumers (T1), while the two other categories (T2 and T3) were based on the median of the PS distribution among consumers in each food category; OB, obese; cal, calories; SD, Standard Deviation. a. % increase in growth was calculated by dividing the B coefficient by the mean growth rate (36 g/wk) and multiplying by 100; b. Overweight was classified as a weight standard deviation score above 1.04, which is above the 85th percentile. A total of 333 children were classified as overweight and 1606 as normal weight according to the UK population mean in 1990 for child's age, sex, and gestational age; c. Logistic regression analyses assessed the odds of being normal weight versus overweight for higher meal size.

**TABLE 2:** Characteristics of the intervention studies investigating food PS and adiposity in children.

Reference	Study population	Total sample	Duration (days)	Intervention	Intervention description	Baseline adiposity	End of intervention adiposity	Brief conclusions
Torbahn et al, 2017^69^	German adolescents (68 f, 63 m) with obesity, aged between 8 and 16 y (*M* = 11.78 ± 1.78)	131	NA; follow‐up after 365 and 720 d	Treatment	Multidisciplinary education. Nutrition modules specifically targeted preferred PS, eating rate, and food choice	BMI‐SDS: 2.30 ± 0.39	∆ BMI‐SDS (1y): ‐ 0.30 ± 0.37 ∆ BMI‐SDS (2‐y): −0.27 ± 0.45	Reduction of ideal PS (preferred PS) was associated with 1‐y (*d* = 0.4) and 2‐y weight reduction (*d* = 0.20)
Loney et al, 2010^76^	Emirati adolescents with obesity aged between 14 and 19 y	15	56	Treatment	4 PS‐controlled (PS served) meals daily offering 12.1 MJ/d and nutritional counseling	BMI 41.3 ± 3.4 (kg/m^2^)	∆ BMI: −4.1 ± 3.0	PS served intervention with nutritional counseling was more effective than counseling alone (*d* = 0.87)
		15	56	Control	4 daily meals where PS was not regulated and nutritional counseling	BMI 36.3 ± 1.0 (kg/m^2^)	∆ BMI: −2.0 ± 1.4	

This table describes the characteristics of the intervention studies included in the review which investigate an index of portion size and a measure of body weight/adiposity in children. Information about the study population, total sample size, duration (days) of intervention, the type of intervention, and details about the intervention itself, together with information about the baseline and end‐of‐intervention adiposity and brief conclusions from two studies are provided. The studies are presented in descending order based on publication date, for example, the study by Torbahn and colleagues (2017) is presented first as it is the most recent. A note is included at the bottom of the table to clarify abbreviations used in the table.

Note. Abbreviations: f, female; m, male; y, years; M, Mean; NA, not available; d, days; PS, portion size; BMI, body‐mass index; SDS, standard deviation scores; ∆, mean change; MJ, megajoules; kg, kilograms; m^2^, meter squared.

Data from 16,830 children were included in the current review. The age of children ranged from 12 months to 20 years, although only two studies included children under the age of three.^43^, ^70^ Eight studies included data from preschool children, aged 3 to 6 years old,^37^, ^42^, ^60^, ^62^, ^64^, ^65^, ^68^, ^73^, ^75^ while the majority (*n* = 19) of studies were conducted in school‐age children^35^, ^37^, ^42^, ^61^, ^64^, ^67^, ^68^, ^69^, ^72^, ^74^, ^75^ or adolescents.^35^, ^37^, ^61^, ^63^, ^66^, ^69^, ^71^, ^76^ In the included studies there was typically a balanced number of male and female participants, although six studies did not report the sex/gender of participants,^43^, ^61^, ^62^, ^69^, ^72^, ^76^ one study included only male participants,^75^ and one study included only female participants.^71^ Most studies reported the sex of the participants,^35^, ^42^, ^60^, ^64^, ^66^, ^67^, ^68^, ^70^, ^73^ while fewer studies reported gender,^37^, ^63^ although both sex/gender were assessed using child or parental reports. Therefore, throughout the current review, sex/gender will be used when referring to differences between male and female participants.

### 3.2 Characteristics of PS and adiposity measures

Ten articles defined PS as the amount of food in grams^35^, ^37^, ^42^, ^43^, ^63^, ^70^, ^73^ or energy (MJ or kcal)^66^, ^68^, ^75^ the child typically consumed (i.e., habitual intake). Four studies investigated the amount of food in grams served to, or self‐served by children.^60^, ^61^, ^64^, ^69^ Meanwhile, for eight studies it was not possible to determine the unit of measurement (grams or energy),^61^, ^62^, ^65^, ^67^, ^69^, ^71^, ^72^, ^74^ but it was possible to establish that they reported the amount of food consumed by the child.^62^, ^65^, ^67^, ^69^, ^71^, ^72^, ^74^ However, it is important to note that studies varied in the type of PS measured (e.g., PS served or consumed, desired/ideal/maximum PS), and in the way PS was obtained; while some studies directly observed children during an eating occasion,^60^, ^62^, ^64^, ^73^, ^74^, ^75^ others used diet diaries^35^, ^37^, ^42^, ^43^, ^66^, ^70^, ^71^, ^72^ or tasks using photographs of food increasing in PS.^61^, ^63^, ^65^, ^67^, ^68^, ^69^ Typically, diet diaries and PS tasks were completed by caregivers or parents. In a few studies, children self‐reported their PS.^35^, ^37^, ^42^, ^61^ In only one study was PS manipulated, where children were offered portion‐controlled meals with an energy deficit of 4.2 MJ per day (which equates to approximately 1000 kcal).^76^

The direct indexes of adiposity used in these studies, included waist and hip circumference,^71^ weight status (e.g., ‘normal weight’ vs overweight),^42^, ^60^, ^65^, ^66^, ^70^, ^73^, ^75^ and an index of fat mass.^61^, ^63^ Most of the included studies (*n* = 13), however, reported a measure of BMI (e.g., BMI, BMI percentile, BMI z‐scores, BMI‐for age, and sex/gender) as an indirect index of adiposity, which was often adjusted for the age and sex/gender of the child.^35^, ^37^, ^60^, ^61^, ^62^, ^63^, ^64^, ^67^, ^68^, ^69^, ^72^, ^74^, ^76^ Four studies only measured body weight without adjusting for height.^39^, ^56^, ^60^, ^69^ In 14 of the included studies, the anthropometric data was recorded by trained staff (e.g., researchers, student assistants) or by healthcare professionals, such as pediatrics or health visitors.^35^, ^60^, ^61^, ^63^, ^64^, ^65^, ^66^, ^68^, ^69^, ^70^, ^71^, ^73^, ^74^ Although, in some of the studies the data were reported by the child or their caregiver.^37^, ^42^, ^43^, ^67^, ^70^, ^75^ Three of the conference abstracts included in the review did not describe in sufficient detail how the height and weight of children were measured.^62^, ^72^, ^76^ The studies identified are summarized in the context of the following aims: (1) associations between food PS and adiposity, and (2) effects of PS interventions on adiposity. As highlighted above, the results of studies that included only a measure of body weight will also be described as a positive control in a separate section. Characteristics of all the studies described above are presented in Table 1.

### 3.3 PS and body weight

A prospective study identified in the review investigated whether meal size (average PS) consumed by 21‐month‐old infants predicted their subsequent weight gain (defined as kg/week).^70^ The infants' weight was recorded at baseline and when the child was between two and five years old. Infant's average consumed meal size was positively associated with their weight at baseline, and for every additional 10 kcal consumed per meal at 21 months, infants weighed 21 g more at follow‐up. Similarly, two cross‐sectional studies and one longitudinal study found positive significant associations between average PS consumed by children and an index of body weight.^43^, ^65^, ^75^ McConahy and colleagues found positive significant associations between average consumed PS z‐scores calculated using 2‐day dietary recalls and body weight percentile in young children (one to two years old) from the USA.^43^ In an observational study of four families, Waxman and Stunkard found that the average PS consumed at home by children with obesity (four to 13 years) was significantly larger compared with the PS consumed by their siblings and peers who had not been classified as obese (classified using their body weight). This was the case even after adjusting for children's height (*d* > 1.0).^75^ Additionally, Mahfida and colleagues found positive significant associations between a mother's estimation of their toddler's average PS consumed, especially PS of vegetables and animal protein, and the child body weight.^65^ However, these associations were no longer statistically significant when body weight was adjusted for the age or height/length of the child (e.g., weight for age, length or height for age, and weight for length or height). These studies support our expectation that consumed portion size would be associated with body weight and further highlight the importance of investigating the association between PS and indexes of adiposity which are adjusted for the height/length of children.

### 3.4 Associations between PS and adiposity

#### 3.4.1 PS and BMI/fat mass index

Most studies (n = 8) reported significant associations between PS of a range of foods and body mass or fat mass index. Three cross‐sectional studies found positive significant associations between preferred PS, consumed PS, and BMI percentile.^37^, ^67^, ^68^ However, the associations tended to be of trivial to small effect size (*d* = 0.002 ‐ *d* = 0.38) and varied according to the age (child age in these studies varied from 3 to 19 years) and sex/gender of participants. In addition, Potter and colleagues identified a caregiver's belief of their child's ideal (*d* = 0.84) and maximum portion size (*d* = 0.62), preferred PS, as relatively stronger predictors of child BMI.

Similar results were reported in other studies for consumed PS, but it was found that associations were dependent on the age and sex/gender of the participants, and food type.^35^, ^62^, ^63^, ^72^ For example, Flieh and colleagues found that the average PS consumed over two days of ‘breakfast cereals’ in adolescents (12.5–17.5 years) was positively associated with BMI in male participants, while the average PS consumed of ‘sweet bakery product’ was negatively associated with BMI and fat mass index (FMI, another indicator for diagnosing obesity, which is obtained by dividing fat mass in kilograms by the square height in meters^77^) in female participants.^63^ This was the case even after adjusting for confounders such as physical activity, total energy intake, and socioeconomic status, but the associations were of trivial to small effect size (*d* = 0.001 – *d* = 0.11).^63^ Two of these studies found that the average PS consumed of foods (e.g., cheese, cream, breakfast cereals with high fiber, biscuits, and buns, cakes, pastries, sausages, canned fruits), and beverages (other milk, carbonated soft drinks) relatively higher in energy density, were positively associated with BMI in British (11–18 years) and Cuban (7–9 years) children.^35^, ^72^ One study in Japanese pre‐school children found that the amount of lunch consumed (consumed PS) was associated with BMI only in children aged 5–6 years old and in those with normal weight, although it is important to note that the sample size for this study was limited (63 children, with 33 children being from the older class). Across these studies, the magnitude of the effect could not be determined. In a prospective intervention, Torbahn and colleagues found that smaller ideal PS of food (preferred PS) in adolescents aged between eight to 16 years old (as reported by their parents) was associated with 1‐year (*d* = 0.472) and 2‐year BMI reduction (*d* = 0.366).^69^

By contrast to the above studies, four studies found no associations between ideal, maximum (preferred PS), self‐served, consumed PS, and BMI/FMI in children.^60^, ^61^, ^64^, ^74^ When Native American children (10–12.6 years) were asked to select their ideal PS of five foods (preferred PS), correlations with child BMI z‐score were trivial to small (*d* = 0.06 ‐ *d* = 0.24).^74^ Similar patterns in results were reported by McGale and colleagues for the associations between child BMI and the PS of cereal self‐served, and consumed by school‐age children in the UK (*d* = 0.106 – *d* = 0.17).^64^ Additionally, Savage and colleagues (2012), offered preschool children (3–5 years) in the USA experimental menus consisting of macaroni cheese (400 g) which were either plated or for self‐serving, and children's subsequent ad libitum consumption was measured.^60^ When looking at the amount of food children self‐served, this was not significantly associated with BMI z‐score (*d* = 0.5). These studies were based on more limited samples (≤ 63), which means that the observations may have lacked the power to detect a meaningful effect. Coxon and colleagues performed a larger study (*N* = 229) looking at the maximum portion size (preferred PS) of five foods (peas, sweetcorn, peanuts, chocolate, and M&M's) that children and adolescents selected on an online computerized task.^61^ Results suggested that there were positive correlations between BMI z‐scores and the maximum portion size of peas, sweetcorn, peanuts, chocolate, and M&Ms selected by children and adolescents, although these were small effects (*d* = 0.18 – *d* = 0.263). The correlations between the relative fat mass index (FMI) and maximum portion size of the five foods were trivial to small (*d* = 0.07 – *d* = 0.2), and only the correlation between FMI and chocolate was positive. In a regression analysis, FMI was not a significant predictor of maximum portion size, when adjusting for age, time since last meal, and eating rate.

Overall, the associations between served, consumed, and preferred PS of foods and BMI/FMI appear to be of small‐moderate effect, and largely dependent on individual characteristics (age, sex/gender) and food type. These studies may still provide some support for preferred PS of food selected by mothers for their children, and PS self‐served or consumed by preschool and school‐aged children being associated with child BMI and FMI.

#### 3.4.2 PS and weight status

Three of the studies discussed above also reported differences in PS self‐served or consumed according to children's weight status (normal weight vs. overweight and obesity, together).^60^, ^63^, ^72^ Savage and colleagues conducted further analyses which revealed that preschool children who were identified as overweight served and consumed larger PS of food (*d* = 0.89).^60^ Additionally, Flieh and colleagues found average PS of ‘carbonated soft and isotonic drink’ consumed was significantly larger in boys with overweight and obesity, while PS of ‘bread and rolls’ and ‘sweet bakery products’ consumed were significantly larger in girls with overweight and obesity.^63^ Similarly, Cabrera and colleagues reported average PS of sausages, cereals, and canned fruits consumed was significantly larger in school children categorized as overweight.^72^

Three other studies reported consistent results for consumed PS.^42^, ^66^, ^73^ Lioret and colleagues found a positive correlation of small‐medium effect between average PS of energy‐dense foods consumed at home (e.g., biscuits, sweetened pastries, liquid dairy products) and weight status in children aged three to six‐year‐old and 7 to 11 year‐old (*d =* 0.17 – *d* = 0.66).^42^ These findings were extended by Lin and colleagues, who reported that PS consumed at home and in childcare settings was associated with child weight status. Nursery children (3.1–6.7 years) who consumed increased PS or “more than needed”, compared to the reference portion size of rice and cooked dishes estimated by researchers, tended to be classified as overweight or obese. However, the associations between PS consumed at home and children's weight status were of small‐medium effect size (*d* = 0.17 – *d* = 0.45).^73^ Tripicchio and colleagues further identified positive associations between adolescent weight status (normal weight, overweight, obese) and average snack size consumed (of beverages, food, and beverages and foods, together), albeit of small effect size (*d* = 0.15 – *d* = 0.23).^66^ Overall, it appears the magnitude of the effect for the associations between served and consumed PS and weight status is small‐medium, consistent with studies that investigated BMI as a continuous measure.

#### 3.4.3 PS and other measures of adiposity

Only one of the cross‐sectional studies identified in the current review investigated the association between ideal PS (preferred PS) and waist and hip circumference.^71^ Adolescent girls who selected larger ideal PS of fast food, had a significantly higher waist and hip circumference. Limited data were available from this study and the magnitude of the effect could not be determined.

### 3.5 Effects of PS interventions on adiposity

Two intervention studies were identified which investigated the effect of reduction of preferred or consumed PS on indexes of adiposity in children (characteristics of these studies are presented in Table 2).^69^, ^76^ The study by Torbahn and colleagues was an observational prospective intervention (before‐after intervention), in which German adolescents with obesity were provided with several educational programs, covering topics such as medical background, nutrition, exercise, and psycho‐social behavior.^69^ The nutrition program included information on how to select and identify the ingredients of food, how to purchase and prepare food, but was specifically targeting PS, eating rate, and food choice. After partial adjustment for confounders (increase of consumption of salad, fruits, vegetables, and whole‐grain bread), analyses revealed that a reduction in the PS typically preferred by children (resulting from educational modules) was associated with a reduction in BMI‐standardized deviation score (BMI‐SDS = BMI adjusted for sex and age) at 1‐year follow‐up and two‐year follow‐up, although the magnitude of these effects were moderate‐small (*d* = 0.4 and *d* = 0.2, respectively). It should be noted that, while anthropometric data were collected by healthcare professionals, PS was assessed using a computerized task completed by parents, which was measuring preferred PS.

Loney and colleagues conducted a non‐randomized controlled trial, in which Emirati adolescents with obesity were allocated in equal numbers to an intervention (BMI at baseline ± SD: 41.3 ± 3.4 kg/m^2^) or control group (BMI at baseline ± SD: 36.3 ± 1.0 kg/m^2^).^76^ In both groups adolescents received nutritional counseling and four meals per day for eight weeks. In the intervention group, adolescents received portion‐controlled meals (reduction of PS served) which provided 12.1 MJ per day, which the authors suggested would provide a daily energy deficit of approximately 4.2 MJ, leading to one‐kilogram reduction in body mass per week. Adolescents in the intervention group lost significantly more weight (∆BMI ± SD: −4.1 ± 3.0 kg) than the control group (∆BMI ± SD: −2.0 ± 1.4 kg), and the association was of large effect size (*d* = 0.87, *p* < 0.05). Based on these findings, Loney and colleagues argued that providing adolescents with nutritional counseling and portion‐controlled meals is more effective in reducing BMI compared to nutritional counseling alone. However, it is important to note that the groups were not matched for BMI at baseline (BMI control group ± SD: 36.3 ± 1.0; BMI intervention group ± SD: 41.3 ± 3.4), which could indicate significant confounding. For example, the intervention group participants may have been more motivated to lose weight than the less overweight comparison group participants. The manipulation of portion size in this study led to a daily energy deficit of 1,000 kcal, which is quite extreme given that an adolescents energy requirements average between 2,200 and 2,800 kcal. Nonetheless, the results of these studies are consistent with a sustained effect of reduced food PS on BMI.

### 3.6 Risk of bias

Five (22.7%) of the identified studies were evaluated as high quality according to the NOS criteria (with a NOS score of ≥ 7).^35^, ^42^, ^63^, ^70^, ^73^ Typically, the selection (e.g., sample representativeness, sample size) and comparability (e.g., adjusting for important confounders) of studies were key methodological limitations. Specifically, 12 studies (54.5%) inadequately justified the sample size, and 14 studies (63.6%) did not sufficiently discuss response rates or comparability between respondents and non‐respondents. Most studies (63.6%) adjusted their models for confounders (e.g., energy density, physical activity or sedentary time, age, sex, socio‐economic status), although only four of these studies adjusted their models for energy density of food and physical activity. A key concern related to the prospective and intervention studies was that in two of the studies, more than 50% of participants were lost at follow‐up (55–60%).^69^, ^70^ Individual scores of each study according to the NOS criteria are presented in the Supporting Information Data S2, Table S3 and Table S4. Only one of the studies was pre‐registered as a Clinical Trial.^73^ Ten studies reported no relevant conflicts of interest (50%),^35^, ^60^, ^63^, ^66^, ^67^, ^68^, ^70^, ^71^, ^73^, ^76^ for nine studies conflicts of interest could not be identified (40.9%)^37^, ^42^, ^43^, ^61^, ^62^, ^65^, ^72^, ^74^, ^75^ and two studies reported conflicts of interest (9.1%).^64^, ^69^

## 4 DISCUSSION

The current paper is the first to systematically review evidence for the association between a PS‐related outcome (served, consumed, or preferred PS) and a direct or indirect index of body adiposity in children, meaning BMI, weight for height, weight status, and other indexes of body fatness. Studies reporting only body weight were still included as a positive control, as we consider body weight to be a relatively ‘trivial’ outcome, especially in cross‐sectional and longitudinal studies. Therefore, the aims of the review were two‐fold; first, we wanted to examine the associations between food PS and measures of body adiposity in children. Second, we wanted to understand if PS interventions (decrease or increase) led to significant and lasting changes in children's body adiposity.

As expected, consumed PS was positively associated with body weight.^39^, ^56^, ^60^, ^69^ However, in the present review we also found PS (served, consumed, or preferred) was associated with measures of body adiposity such as BMI/FMI,^35^, ^37^, ^60^, ^62^, ^63^, ^64^, ^67^, ^68^, ^72^, ^74^ and waist and hip circumference.^71^ Additionally, children with overweight and/or obesity typically selected and consumed larger PS of food compared to those identified as healthy weight.^42^, ^59^, ^60^, ^63^, ^66^, ^72^, ^73^ Effect sizes were typically small. Across a range of designs and outcome measures, the observed effect size differed based on the sex/gender of the participants or based on the food type investigated (high‐energy dense food vs low‐energy‐dense food). However, there was no consistent pattern to these findings. Importantly, most studies included in the current review investigated the average PS consumed by children.^35^, ^37^, ^42^, ^43^, ^63^, ^66^, ^70^, ^72^, ^75^ Four studies measured the PS self‐served and/or consumed by children.^60^, ^62^, ^64^, ^67^, ^73^ Within these, only two studies, which were experimental or quasi‐experimental, investigated the amount of food children self‐served.^60^, ^64^ Compared to studies which investigated the average PS consumed, the effect size in studies that investigated actual PS consumed or served was trivial to medium (*d* = 0.1–0.5). This was with the exception of one study by Savage and colleagues, where a large effect was reported for the relationship between PS self‐served and weight status (*d* = 0.89). Finally, seven studies provided an estimation of the preferred PS (ideal or maximum portion size) a child would consume (either reported by the child or a caregiver).^61^, ^65^, ^68^, ^69^, ^71^, ^74^ The effect size in these studies also varied significantly, from trivial to large (*d =* 0.012–0.84). In particular, there was a trend for parent‐estimated PS rather than child‐reported PS to be more highly associated with child BMI (for example in the papers by Potter and colleagues, and Torbahn and colleagues).

Some of these studies, but not all, identified a trend for children with overweight and/or obesity to select and consume larger PS of food compared to children who were classified as “healthy weight”. When differences in PS were investigated between children considered of ‘healthy weight’ and those identified as overweight and/or obese, the magnitude of the effect was larger. Although there is some prospective evidence to suggest PS consumed and served is associated with adiposity in children, no cause and effect could be established. It is not clear if PS served or consumed contributed to an increase in adiposity in children, or whether children with higher adiposity, due to an inherited tendency toward higher adiposity, served or consumed larger PS of food, as the evidence relies on cross‐sectional or longitudinal correlations. Alternatively, or as well, these associations could be explained by other factors that could precede consumption (e.g., increased availability of more energy‐dense foods) or happen during consumption (e.g., receiving encouragement to finish all the food on the plate).

Only two intervention studies were identified, both of which suggested a reduction in ideal or served PS led to a subsequent reduction in BMI in eight to 19‐year‐olds with overweight and obesity. However, these interventions were not randomized, only one had a control group, and there was no evidence in younger children. When food PS is reduced, it is expected that total energy intake is also lowered, and if this is maintained over a long enough timeframe BMI reduction is expected.^45^ However, the effect of food PS on total energy intake and body adiposity is also reliant on how often individuals consume food throughout the day (i.e., eating frequency), and how active children are (sedentary behavior or physical activity).^43^ This is why it would be important to investigate the effect of PS on adiposity while considering energy density, eating frequency, and physical activity of children in future randomized controlled trials.

Several studies identified during the searches did not meet the inclusion criteria but provided interesting insights into the association and relationship between PS and measures of adiposity in children. Five studies were excluded because the measures of body adiposity were only included in statistical models as covariates and no direct relationship between PS and adiposity was reported.^20^, ^32^, ^78^, ^79^, ^80^ One of these studies found that children with higher BMI percentile (adjusted for age and sex) increased their energy intake to a greater extent when exposed to larger PS of food.^32^ On the contrary, other studies found that the association between PS and energy intake did not vary according to children's weight status or BMI.^19^, ^78^, ^79^, ^80^ A further study was excluded during data extraction because only the volume of food (ml), rather than portion size (g or kcal) was included in the analysis. This paper found that the BMI percentile was not a significant predictor of the portion size in ml self‐served, or consumed by children.^81^

## 5 LIMITATIONS

While 20 studies were identified exploring the association between a measure of PS and an index of adiposity in children, the current knowledge is limited by the small number of studies addressing our second research question, namely the effect of PS interventions on adiposity (*n* = 2). Due to the high heterogeneity in the measures and outcomes related to PS and adiposity, and the lack of sufficient data to estimate an effect size for each included study, a composite score could not be obtained. It is also worth noting that anthropometric and PS measures were typically reported by parents, which raises potential concerns about the accuracy and reliability of the data.^82^, ^83^ While most studies suggested there is a positive association between PS served, consumed, or preferred and adiposity in children, the findings of the current review could also be impacted by research reporting and publication bias,^84^, ^85^, ^86^ and may be limited due to the exclusion of articles that were not available in English.

## 6 FUTURE DIRECTIONS

To address these concerns, we propose several factors which need further consideration. First, there was much variation in the unit used to measure food PS (grams or kcal), and which constructs were assessed (e.g., average PS, ideal PS, typical PS, served PS, consumed PS). When discussing PS as the grams of food consumed or served by an individual, observations may be confounded by the energy density of the food.^87^ For example, a previous study found that individuals consumed 56% more energy (221 kcal) when they were offered the largest portions of higher energy‐dense foods, compared to when they were served the smallest portions of low energy‐dense foods.^88^ Therefore, it may be worthwhile to consistently refer to PS as the amount of food in calories selected or consumed by an individual on a single eating occasion, but also consider the individual foods that make up the meals consumed by children.

Additionally, most research to date on the portion size effect in children has highlighted the amount of food children are served as an important predictor of their consumption.^22^, ^32^ A survey of 1,000 UK mothers carried out by the Infant and Toddler Forum revealed that 79% of parents typically offered their children larger portions than those recommended. In addition, a qualitative study showed that in a sample of parents of one‐to‐two‐year‐olds, most were unaware of existing portion size guidance.^89^ As a result, research has begun to focus on how parents make decisions about the amount of food to serve their children. Several studies have suggested that parents may use their own portion size as a guide, either by offering children half of an adult portion or giving everyone in the family the same amount of food.^89^, ^90^, ^91^, ^92^ Experimental studies have also identified moderate‐strong positive associations between the amount of food parents served themselves and the amount of food they served to their children.^93^, ^94^, ^95^ In addition, one of the included studies by Potter and colleagues found that parents' estimate of child's ideal portion size was a significant predictor of their child's BMI.^68^ In the present review, the majority of evidence focused on the relationship between actual or average portion size consumed by children, which was surprising given the large body of literature highlighting the importance of portioning practices for parents.^96^ While the amount of food served typically correlates with the amount of food consumed in children,^19^, ^26^ this relationship is not perfectly linear, and it is very common for children to leave some food on the plate or even ask for seconds. As a result, to better disentangle the relationships between the amount of food children are served, the amount they consume, and their subsequent adiposity, more research is required which incorporates both measures of PS.

Second, there are concerns about the body weight and adiposity measures used in the included studies. Four studies only reported the body weight or body weight change in association with portion size, which as we highlighted above would be a “trivial” outcome. The rest of the studies tended to include BMI or BMI‐percentile as an index of adiposity in children. BMI is a popular measure of body composition and a practical instrument for tracking population health.^97^ Compared to other indirect indicators of body adiposity (e.g., BMI z‐scores), BMI and BMI percentiles are considered good alternatives for tracking changes in adiposity in children.^98^ However, several concerns have been highlighted about the use of BMI as an indicator of obesity in children. One study suggested that appropriate equipment, measurement protocols, and regular training are necessary to ensure anthropometric data (height, weight) are accurate, highlighting significant concerns about the use of self‐report or parental reports of height and weight.^99^ Additionally, other studies that aimed to compare BMI with other anthropometric measures (e.g., triceps skinfold, fat mass), found that BMI can be used to screen or identify children with obesity, but it is less accurate at identifying children with overweight.^100^ Other authors argue that BMI z‐scores are often misclassified and may not be appropriate when investigating changes in adiposity over time.^98^, ^101^, ^102^ More appropriate measures of adiposity (e.g., waist‐hip circumference, body composition), and not just body weight, which are used consistently across studies are needed to determine whether PS is a risk factor for adiposity. Such measures should be included in future interventions or trials alongside BMI.

Third, the findings of the current review mostly rely on observational evidence from cross‐sectional, cohort, and case studies, as only two interventions (non‐randomized) were identified. Even though we would expect that in the absence of compensation, a manipulation of PS would impact the energy intake of an individual, and hence their body adiposity, more high‐quality evidence is necessary before concluding about causality. Ideally, a randomized controlled trial might be performed where children with overweight or obesity are recruited and allocated to either a control (offered free food where PS is not manipulated) or treatment group (offered free food where served PS is manipulated). However, there are several concerns or considerations related to the running of such studies: a. How will subjects be selected (e.g., age, level of adiposity)?; b. How will PS be manipulated (e.g., one, two, or three eating occasions)?; c. How long will PS be manipulated (e.g., one week, one month, one year)?; d. Which foods should be manipulated (e.g., all foods, foods high in fat and sugar, energy‐dense foods)?; e. Potential adverse outcomes (e.g., affecting growth of children, undernutrition)?

A key consideration is how a reduction in PS can be implemented at a population‐level to potentially reduce child overweight and obesity rates. A recent study found that variations in PS were more likely to be attributed to differences across eating occasions (i.e., children consumed larger portions of foods at certain times throughout the day), and not differences between children (i.e., children did not consume significantly larger portions compared to their peers). The differences in PS across eating occasions were explained by characteristics of the environment. For example, PS of eating occasions were larger in eateries, nurseries, and at home if the food was consumed with family or friends, in front of the TV, or while sitting at a table.^103^ Since PS of foods consumed by children vary more within the child and are larger outside of the home environment (where presumably the served PS is larger), interventions or recommendations for PS reduction could target these environments (i.e., restaurants and nurseries). Additionally, previous research has suggested several PS ‘downsizing’ strategies for children and their families, such as a. Adjusting proportions of foods within a meal to encourage reduced consumption of higher energy‐dense foods (smaller portions offered), and increased consumption of fruits and vegetables (larger portions offered); b. Using packaging strategies to control PS or provide portion control aids; c. Addressing social norms to influence dietary intake.^104^, ^105^

Most of the evidence to date has focussed on evaluating the effectiveness of PS replacement, rather than reduction. Indeed, Ello‐Martin and colleagues identified numerous studies that showed that consuming portions of foods lower in energy density (e.g., fruits, vegetables) promotes a reduction in energy intake (while maintaining levels of fulness), and maybe a more successful weight loss strategy than reducing the weight/volume of food served.^106^ Furthermore, a clinical trial that investigated two strategies for PS control of snacks in preschool children found that compared to snack reduction (i.e., providing half a portion at snacking occasions), snack replacement (i.e., replacing snacks high in energy density with snacks of lower energy density, while maintaining the usual amount and schedule of snacking) had a more positive impact on children's diets (consumption of vegetables increased, and daily energy intake decreased) and was better received by mothers. This may suggest that environments in which children consume larger PS, such as restaurants and nurseries, should be encouraged to adopt ‘downsizing’ PS strategies, such as food replacement (e.g. replacing energy‐dense restaurant side dishes or snacks served in childcare with fruit and vegetables).

## 7 CONCLUSIONS

While an association between served, consumed, and preferred PS and adiposity in children may be established, evidence relies on observational research. Before recommending PS interventions, such as reduction of served PS as a strategy to prevent or reduce overweight and obesity in children, better quality evidence is needed to establish causal pathways and the contribution of other predictors of dietary intake (energy density of foods, eating frequency), and energy balance (physical activity or sedentary behavior).

## CONFLICT OF INTEREST STATEMENT

No conflicts of interest were declared.

## Supporting information


**Data S1.** This spreadsheet shows the detailed information that was extracted for each of the included studies for the review.

**Data S2.** This file includes further supporting information, such as PRISMA flow diagrams, examples of search terms, and information about the quality assessment of included studies. Detailed captions for each Table and Figure included in the Supplementary Material are presented here.
