# Supplementary material for: A systematic review of the relationship between portion size and indexes of adiposity in children
Source: Obes Rev. 2025 Apr 27;26(8):e13928. doi: 10.1111/obr.13928 (PMC12246891; doi:10.1111/obr.13928)
Supplement: Supplementary file 2 — Data S2. This file includes further supporting information, such as PRISMA flow diagrams, examples of search terms, and information about the quality assessment of included studies. Detailed captions for each Table and Figure included in the Supplementary Material are presented here. [file OBR-26-e13928-s002.pdf]

A systematic review of the relationship between portion size and indexes of adiposity in children

Anca T. Dobrescu[1], Alice Porter[2,3], Danielle Ferriday[1,3], Peter J. Rogers[1,3]

[1] Nutrition and Behaviour Unit, School of Psychological Science, University of Bristol, Bristol, United Kingdom

[2] Population Health Sciences, Bristol Medical School, University of Bristol, Bristol, United Kingdom

[3] NIHR Bristol Biomedical Research Centre, University Hospitals Bristol and Weston NHS Foundation Trust and University of Bristol, United Kingdom

Correspondence: Anca Dobrescu, Nutrition and Behaviour Unit, School of Psychological Science, University of Bristol, Bristol, BS8 1TU, United Kingdom. Email: anca.dobrescu@bristol.ac.uk.

Supporting Information 1 – PRISMA writing checklist

Table S1. PRISMA writing checklist

Section/topic	#	Checklist item	Reported on page #
**TITLE**			
Title	1	Identify the report as a systematic review, meta-analysis, or both.	1
**ABSTRACT**			
Structured summary	2	Provide a structured summary including, as applicable: background; objectives; data sources; study eligibility criteria, participants, and interventions; study appraisal and synthesis methods; results; limitations; conclusions and implications of key findings; systematic review registration number.	4
**INTRODUCTION**			
Rationale	3	Describe the rationale for the review in the context of what is already known.	5-8
Objectives	4	Provide an explicit statement of questions being addressed with reference to participants, interventions, comparisons, outcomes, and study design (PICOS).	8
**METHODS**			
Protocol and registration	5	Indicate if a review protocol exists, if and where it can be accessed (e.g., Web address), and, if available, provide registration information including registration number.	8
Eligibility criteria	6	Specify study characteristics (e.g., PICOS, length of follow-up) and report characteristics (e.g., years considered, language, publication status) used as criteria for eligibility, giving rationale.	8-12
Information sources	7	Describe all information sources (e.g., databases with dates of coverage, contact with study authors to identify additional studies) in the search and date last searched.	9-12
Search	8	Present full electronic search strategy for at least one database, including any limits used, such that it could be repeated.	Supporting Information 2, Table S2
Study selection	9	State the process for selecting studies (i.e., screening, eligibility, included in systematic review, and, if	10-11

		applicable, included in the meta-analysis).	
Data collection process	10	Describe method of data extraction from reports (e.g., piloted forms, independently, in duplicate) and any processes for obtaining and confirming data from investigators.	~~10-11~~
Data items	11	List and define all variables for which data were sought (e.g., PICOS, funding sources) and any assumptions and simplifications made.	~~10-11~~
Risk of bias in individual studies	12	Describe methods used for assessing risk of bias of individual studies (including specification of whether this was done at the study or outcome level), and how this information is to be used in any data synthesis.	~~11-12~~
Summary measures	13	State the principal summary measures (e.g., risk ratio, difference in means).	~~12~~
Synthesis of results	14	Describe the methods of handling data and combining results of studies, if done, including measures of consistency (e.g., I^2) for each meta-analysis.	~~12~~
Risk of bias across studies	15	Specify any assessment of risk of bias that may affect the cumulative evidence (e.g., publication bias, selective reporting within studies).	N/A
Additional analyses	16	Describe methods of additional analyses (e.g., sensitivity or subgroup analyses, meta-regression), if done, indicating which were pre-specified.	N/A
**RESULTS**			
Study selection	17	Give numbers of studies screened, assessed for eligibility, and included in the review, with reasons for exclusions at each stage, ideally with a flow diagram.	~~12-13~~
Study characteristics	18	For each study, present characteristics for which data were extracted (e.g., study size, PICOS, follow-up period) and provide the citations.	~~14-16~~
Risk of bias within studies	19	Present data on risk of bias of each study and, if available, any outcome level assessment (see item 12).	~~39~~, Supporting Information ~~4 & 5~~
Results of individual studies	20	For all outcomes considered (benefits or harms), present, for each study: (a) simple summary data for each intervention group (b) effect estimates and confidence intervals, ideally with a forest plot.	~~12-39~~
Synthesis of results	21	Present results of each meta-analysis done, including confidence intervals and measures of consistency.	N/A

Risk of bias across studies	22	Present results of any assessment of risk of bias across studies (see Item 15).	N/A
Additional analysis	23	Give results of additional analyses, if done (e.g., sensitivity or subgroup analyses, meta-regression [see Item 16]).	N/A
**DISCUSSION**			
Summary of evidence	24	Summarize the main findings including the strength of evidence for each main outcome; consider their relevance to key groups (e.g., healthcare providers, users, and policy makers).	39-41
Limitations	25	Discuss limitations at study and outcome level (e.g., risk of bias), and at review-level (e.g., incomplete retrieval of identified research, reporting bias).	41
Conclusions	26	Provide a general interpretation of the results in the context of other evidence, and implications for future research.	42-45
**FUNDING**			
Funding	27	Describe sources of funding for the systematic review and other support (e.g., supply of data); role of funders for the systematic review.	1

Note. Table obtained from Moher D, Liberati A, Tetzlaff J, Altman DG, The PRISMA Group (2009). Preferred Reporting Items for Systematic Reviews and Meta-Analyses: The PRISMA Statement. PLoS Med 6(6): e1000097. doi:10.1371/journal.pmed1000097

Supporting Information 2 – Example search strategy

Table S2. Example search strategy used in Ovid Medline based on the PICO framework

Line Number	*Search Term*
1	exp Infant, Newborn/ or exp Child, Preschool/ or exp Infant/ or exp Child/
2	(baby or babies).tw.
3	(newborn* or "infant, newborn*" or "newborn infant*").tw.
4	(infant* or infancy).tw.
5	child*.tw.
6	("preschool child*" or "child*, preschool").tw.
7	preschooler*.tw.
8	toddler*.tw.
9	“school child*”.tw.
10	exp adolescent/
11	adolescen*.tw.
12	teen*.tw.
13	youth*.tw.
14	exp portion size/ or exp serving size/
15	“portion* size*”.tw.
16	“food* portion*”.tw.
17	“portion* of food”.tw.
18	“serving* size*”.tw.
19	exp body weight/ or exp body mass index/ or exp body weight changes/ or exp weight gain/ or exp weight loss/ or exp obesity/ or exp pediatric obesity/ or exp obesity, abdominal/ or exp waist circumference/ or exp adipose tissue/ or exp waist hip ratio/
20	“body weight”.tw.
21	“weight, body”.tw.
22	“body mass index”.tw.
23	BMI.tw.
24	“body mass index percentile”.tw.
25	“BMI percentile”.tw.

26	“Body mass index for age percentile”.tw.
27	“BMI for age growth chart*”.tw.
28	“Body mass index for age percentile*”.tw.
29	“Body mass index for age growth chart*”.tw.
30	“BMI-SDS”.tw.
31	“body mass index standard deviation score*”.tw.
32	“BMI standard deviation score*”.tw.
33	“body fat”.tw.
34	“weight status”.tw.
35	“body composition”.tw.
36	“weight gain”.tw.
37	“weight loss”.tw.
38	“body weight change*”.tw.
39	adipos*.tw.
40	overweight.tw.
41	“over weight”.tw.
42	obes*.tw.
43	“waist hip ratio”.tw.
44	“waist circumference”.tw.
45	slim*.tw.
46	“BMI change”.tw.
47	“BMI classification”.tw.
48	1 or 2 or 3 or 4 or 5 or 6 or 7 or 8 or 9 or 10 or 11 or 12 or 13
49	14 or 15 or 16 or 17 or 18
50	19 or 20 or 21 or 22 or 23 or 24 or 25 or 26 or 27 or 28 or 29 or 30 or 31 or 32 or 33 or 34 or 35 or 36 or 37 or 38 or 39 or 40 or 41 or 42 or 43 or 44 or 45 or 46 or 47
51	48 and 49 and 50
52	limit 51 to (humans, english language and yr="2022 -Current")

Note. Ovid Medline 1806 to April 9 April 2024, search ran on 10 April 2024 which obtained 35 results using search in title and abstract and limited to English, human research, and 2022-current.

Supporting Information 3 – PRISMA flow diagrams for updated searches

Figure S1. PRISMA flow diagram presenting the identification of resources from the updated searches on 18 August 2022

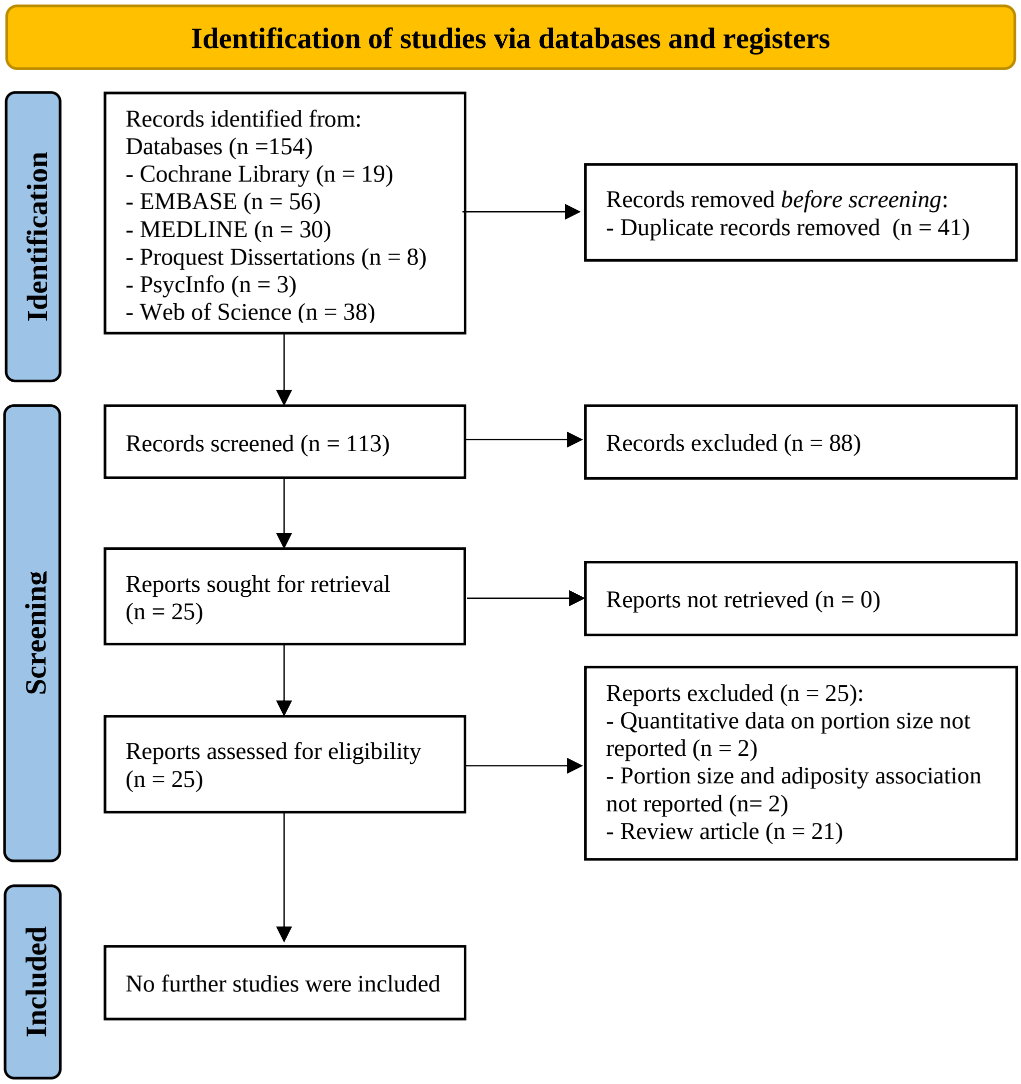

Figure S2. PRISMA flow diagram presenting the identification of resources from the updated searches on 10 April 2024

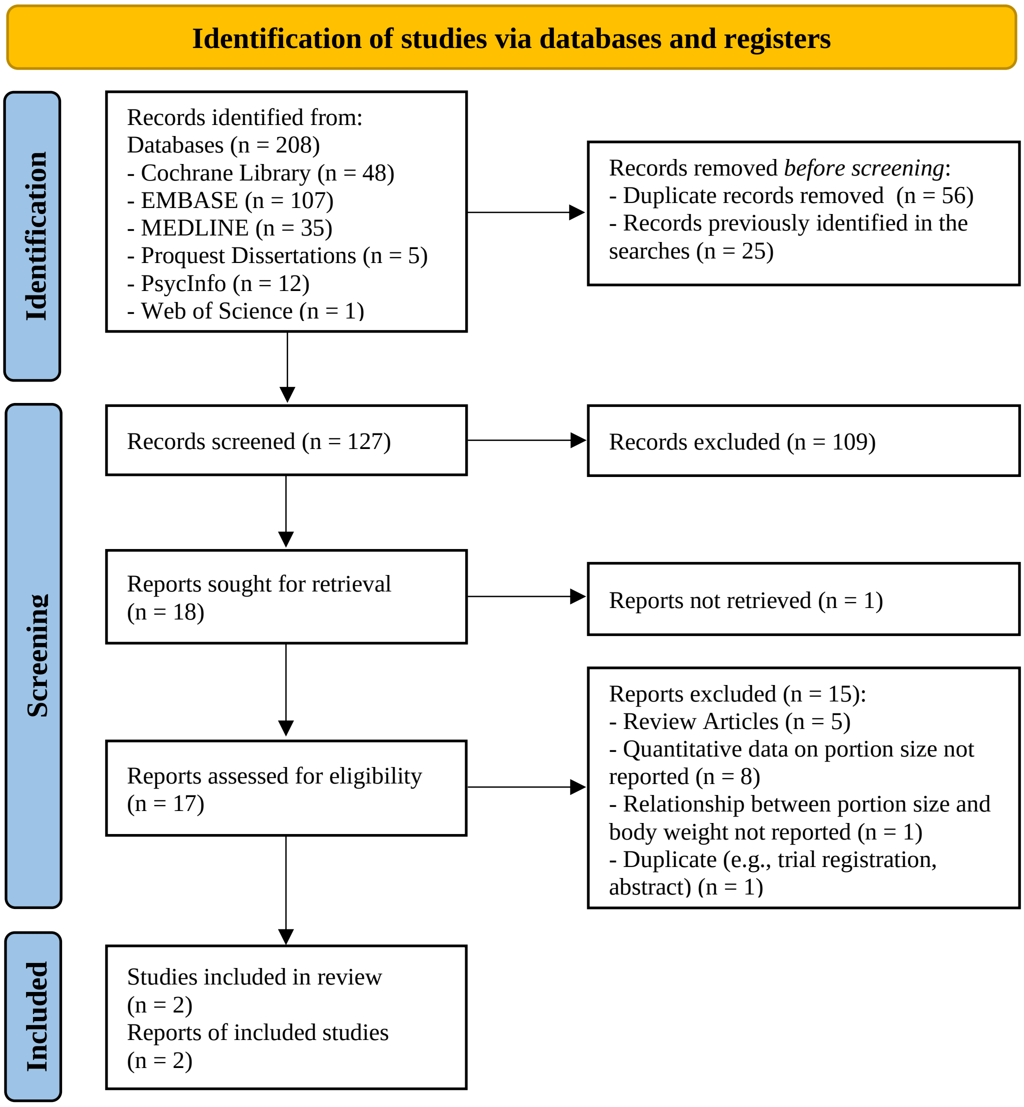

Supporting Information 4 - Newcastle Ottawa Scale for Assessing Risk of Bias

Newcastle Ottawa Scale was adapted for the current review to assess the quality of cross-sectional, longitudinal and intervention studies. In total a study can be allocated a maximum of 9 stars.

Selection (maximum of 4 stars can be awarded)

1. Representativeness of the cohort:
   A. Truly representative of the general population (all subjects or random sampling)*
   B. Somewhat representative of the target population (non-random sampling)*
   C. Selected group of participants (e.g., volunteers, nurses)
   D. No description of sampling strategy
2. Sample Size:
   A. Justified and satisfactory*
   B. Not justified
3. Non-respondents:
   A. Comparability between respondents and non-respondents characteristics is established, and the response rate is satisfactory *
   B. Response rate is unsatisfactory, or comparability between respondents and non-respondents is unsatisfactory
   C. No description of response rate or the characteristics of respondents and non-respondents
4. Ascertainment of exposure (portion size):
   A. Researcher measured*
   B. Self or parent report (with extra validation/ reliability information reported for specific target sample)*
   C. Self or parent-report
   D. No description of measurement tool

Comparability (maximum of 2 stars)

The subjects in different outcome groups are comparable, based on the study design or analysis.

1. The study controlled for confounding (e.g., energy density, physical activity, sedentary time, sex, age, socioeconomic status):
   A. Key confounding factors are controlled for (i.e., energy density, physical activity, sedentary time)**
   B. Study controls for other additional factors, such as sex, age, socioeconomic status*
   C. No confounders are controlled for

Outcome/Exposure (maximum of 3 stars for cross-sectional studies)

1. Assessment of outcome (adiposity)
   A. Clinical/Researcher assessment**
   B. Record linkage**
   C. Self or parent-report
2. Statistical test
   A. The statistical test used to analyse the data is clearly described and appropriate, and the measurement of the association is presented, including a measure of effect size and the probability level (p value)*
   B. The statistical test is not appropriate, not described or incomplete.

! For intervention/longitudinal studies the outcome/exposure was assessed differently, although a maximum of 3 stars could be achieved:

Outcome/Exposure (maximum of 3 stars for intervention or prospective studies)

1. Assessment of outcome (adiposity)
   A. Clinical/Researcher assessment*
   B. Record linkage*
   C. Self or parent-report

2. Was follow-up long enough for outcomes to occur (4 weeks based on previous study by Higgins and colleagues, 2022) ?
   A. Yes*
   B. No
3. Adequacy of follow up of cohorts
   A. Complete follow up - all subjects accounted for*
   B. Subjects lost to follow-up unlikely to introduce bias (small number lost) - > 80% follow up, or description provided of those lost*
   C. Follow up rate < 80 % and no description of those lost
   D. No statement

! Follow-up rate was based on the cut-off used in Evidence-Based Medicine:

https://www.cebm.ox.ac.uk/resources/levels-of-evidence/ocebm-levels-of-evidence

NOS modifications

The Newcastle-Ottawa Quality Assessment Scale was adapted for the current review to perform a quality assessments of cross-sectional, longitudinal and intervention studies. The NOS evaluates three key domains of study quality: a. Selection of the cohort; b. comparability of study analysis; c. ascertainment of the outcome. The three quality domains are rated using a star scoring system (see additional information about scoring procedure below) and the summation of the stars is used to generate the total score (maximum 10 stars). A NOS score of $\geq$7 is considered a “good” study, and this was used as a cut-off to classify good study quality (McPheeters et al. 2012).

Supporting Information 5 – NOS Risk of Bias Assessment of Included Studies

Table S3. Newcastle-Ottawa quality assessment scale for cross-sectional studies

The Newcastle-Ottawa Quality Assessment Scale was adapted for the current review to perform a quality assessments of cross-sectional, longitudinal and intervention studies. The NOS evaluates three key domains of study quality: a. Selection of the cohort; b. comparability of study analysis; c. ascertainment of the outcome. The three quality domains are rated using a star scoring system (see additional information about scoring procedure above) and the summation of the stars is used to generate the total score (maximum 10 stars). A NOS score of ≥7 is considered a "good" study, and this was used as a cut-off to classify good study quality (McPheeters et al. 2012)**.**

	**Selection**				**Comparability**	**Outcome**		
**Cross-sectional (n=18)**	Representativeness of the sample	Sample size	Non-respondents	Ascertainment of exposure	Controls for most important confounder	Assessment of outcome	Statistical test	**Total Score**
Coxon et al., 2023[1]	B*	B	C	C	B*	A**	B	4
Yamada et al., 2023[1]	C	B	C	A*	C	A**	B	3
Flieh et al., 2021	B*	A*	A*	B*	A**	A**	A*	9
McGale et al., 2020	C	A*	B	A*	B*	A**	B	5

Mahfida et al., 2019	C	B	C	B*	C	B**	B	3
Tripicchio et al., 2019	A*	B	B	B*	A**	A**	B	6
Godefroy et al., 2018	A*	A*	A*	B*	B*	C	B	5
Potter et al., 2018	B*	B	B	B*	C	A**	A*	5
ALFaris et al., 2015	A*	B	B	B*	C	A**	B	4
Albar et al., 2014	A*	A*	A*	A*	B*	A**	A*	8
Cabrera et al., 2013[1]	C	B	C	B*	C	C	B	1
Lin et al., 2013	A*	A*	A*	A*	B*	A**	A*	8
Savage et al., 2012	C	B	B	A*	B*	A**	A*	5
Lioret et al., 2009	A*	A*	A*	B*	A**	C	A*	7

Huang et al., 2004	A*	A*	A*	B*	B*	C	A*	6
Rodgers, 2004	C	B	C	A*	C	A**	A*	4
McConahy et al., 2002	A*	A*	A*	B*	C	C	B	4
Waxman et al., 1980	C	B	C	A*	B*	A**	A*	5

Note. [1.] These studies scored lower on the NOS due to the fact that only abstracts were available, which typically included limited information about the selection of participants, measurement of outcomes and statistical tests used.

Table S4. Newcastle-Ottawa quality assessment scale for intervention and longitudinal studies

The Newcastle-Ottawa Quality Assessment Scale was adapted for the current review to perform a quality assessments of cross-sectional, longitudinal and intervention studies. The NOS evaluates three key domains of study quality: a. Selection of the cohort; b. comparability of study analysis; c. ascertainment of the outcome. The three quality domains are rated using a star scoring system (see additional information about scoring procedure above) and the summation of the stars is used to generate the total score (maximum 10 stars). A NOS score of ≥7 is considered a "good" study, and this was used as a cut-off to classify good study quality (McPheeters et al. 2012).

	**Selection**				**Comparability**	**Outcome**			
**Intervention / Longitudinal (*n*=3)**	Representativeness of the sample	Sample size	Non-respondents	Ascertainment of exposure	Controls for most important confounder	Assessment of outcome	Duration of follow-up	Adequacy of follow-up	**Total Score**
**Syrad et al., 2016**	A*	A*	A*	B*	B*	A*	A*	B*	8
**Torbahn et al., 2017**	C	B	B	B*	B*	A*	A*	B*	5
**Loney et al., 2010[1]**	C	B	C	A*	C	N/A	A*	A*	3

Note. [1]. This study scored lower on the NOS due to the fact that only the abstract were available, which typically included limited information about the selection of participants, measurement of outcomes and statistical tests used.

Table and Figure Legend

Table S1: This table is the PRISMA writing checklist, which clearly identifies where relevant information can be found within the main manuscript.

Table S2: This table provides an example search strategy for MEDLINE which was used in the search performed in April 2024.

Table S3: This table provides information about the quality assessment (performed using the Newcastle-Ottawa quality assessment scale) of cross-sectional studies. The Newcastle-Ottawa Quality Assessment Scale was adapted for the current review to perform a quality assessments of cross-sectional, longitudinal and intervention studies. The NOS evaluates three key domains of study quality: a. Selection of the cohort; b. comparability of study analysis; c. ascertainment of the outcome. The three quality domains are rated using a star scoring system (see additional information about scoring procedure above) and the summation of the stars is used to generate the total score (maximum 10 stars). A NOS score of >7 is considered a "good" study, and this was used as a cut-off to classify good study quality (McPheeters et al. 2012).

Table S4: This table provides information about the quality assessment (performed using the Newcastle-Ottawa quality assessment scale) for intervention and longitudinal studies. The Newcastle-Ottawa Quality Assessment Scale was adapted for the current review to perform a quality assessments of cross-sectional, longitudinal and intervention studies. The NOS evaluates three key domains of study quality: a. Selection of the cohort; b. comparability of study analysis; c. ascertainment of the outcome. The three quality domains are rated using a star scoring system (see additional information about scoring procedure above) and the summation of the stars is used to generate the total score (maximum 10 stars). A NOS score of >7 is considered a "good" study, and this was used as a cut-off to classify good study quality (McPheeters et al. 2012).

Figure S1: This figure represents the PRISMA flow diagram showing the identification of resources for the systematic review for the second search which was performed in August 2022. The figure shows how many records were identified in each of the databases used for the searches (Cochrane Library, EMBASE, MEDLINE, Proquest Dissertations, PsycInfo and Web of Science). It also demonstrates how many records remained after abstract and full-text screening, and there is detailed information about the reason for exclusion of studies. Finally, the figure identifies the number studies included in the review.

Figure S2: This figure represents the PRISMA flow diagram showing the identification of resources for the systematic review for the second search which was performed in April 2024. The figure shows how many records were identified in each of the databases used for the searches (Cochrane Library, EMBASE, MEDLINE, Proquest Dissertations, PsycInfo and Web of Science). It also demonstrates how many records remained after abstract and full-text screening, and there is detailed information about the reason for exclusion of studies. Finally, the figure identifies the number studies included in the review.
